# ESPO/ Health Sciences Section Symposium: Leveraging Older Adults’ Perceptions of Chronic Illness to Improve Care

**DOI:** 10.1093/geroni/igaa057.2815

**Published:** 2020-12-16

**Authors:** Anyah Prasad, Brianna Morgan, Mary Naylor

## Abstract

In advanced years of life comorbidity of chronic illnesses is a common phenomenon. While chronic illnesses have been documented to impact overall quality of life, morbidity, and mortality, older adults may develop psychological resources in the years of coping and managing their illnesses. These psychological resources can be influenced by individual perceptions and context as people age, and include concepts such as psychological resilience, inner strength, subjective age, and illness perception. This symposium brings together emerging health science scholars’ work on exploring and leveraging these psychological resources to improve care. Amy Ketcham will present a systematic review of psychological resilience and depression in adults with cardiac disease. Brianna Morgan will present a concept analysis exploring the nature of inner strength in people aging with serious illness and cognitive impairment. Anyah Prasad will present results examining the association between chronic illnesses and subjective age and discuss its clinical relevance. Eleanor Rivera will explore illness perception phenotypes in a longitudinal cohort study of older adults with chronic kidney disease. Together, the perspectives shared in this symposium improve understanding of and indicate ways to move toward person-centered and contextual clinical models of care in the management of chronic illnesses among older adults. In addition, the discussant will engage in a dynamic conversation about psychological resources in later life and the role these projects have played in advancing the presenters along their academic trajectories.

